# Commentary of the SKLM to the EFSA opinion on risk assessment of N-nitrosamines in food

**DOI:** 10.1007/s00204-024-03726-1

**Published:** 2024-04-04

**Authors:** Gerhard Eisenbrand, Andrea Buettner, Patrick Diel, Bernd Epe, Petra Först, Tillman Grune, Dirk Haller, Volker Heinz, Michael Hellwig, Hans-Ulrich Humpf, Henry Jäger, Sabine Kulling, Alfonso Lampen, Marcel Leist, Angela Mally, Doris Marko, Ute Nöthlings, Elke Röhrdanz, Joachim Spranger, Pablo Steinberg, Stefan Vieths, Wim Wätjen, Jan G. Hengstler

**Affiliations:** 1 Kühler Grund 48/1, 69126 Heidelberg, Germany; 2 Chair of Aroma and Smell Research, Friedrich-Alexander-Universität Erlangen-Nürnberg, Henkestrasse 9, 91054 Erlangen, Germany; 3 Fraunhofer Institute for Process Engineering and Packaging IVV, Giggenhauser Strasse 35, 85354 Freising, Germany; 4 Department of Molecular and Cellular Sports Medicine, Institute of Cardiovascular Research and Sports Medicine, German Sport University Cologne, Am Sportpark Müngersdorf 6, 50933 Cologne, Germany; 5 Institute of Pharmaceutical and Biomedical Sciences, University of Mainz, Staudingerweg, 55128 Mainz, Germany; 6 Food Process Engineering, TUM School of Life Sciences, Technical University of Munich, Weihenstephaner Berg 1, 85354 Freising, Germany; 7 German Institute of Human Nutrition Potsdam-Rehbrücke (DIfE), Arthur-Scheunert-Allee 114-116, 14558 Nuthetal, Germany; 8 Chair of Nutrition and Immunology, Technical University of Munich, Gregor-Mendel-Strasse 2, 85354 Freising, Germany; 9 ZIEL Institute for Food and Health, Technical University of Munich, Weihenstephaner Berg 1, 85354 Freising, Germany; 10 DIL German Institute of Food Technology, Professor-von-Klitzing-Strasse 7, 49610 Quakenbrück, Germany; 11 Chair of Special Food Chemistry, Technical University Dresden, Bergstrasse 66, 01062 Dresden, Germany; 12 Institute of Food Chemistry, University of Münster, Corrensstrasse 45, 48149 Münster, Germany; 13 University of Natural Resources and Life Sciences, Gregor-Mendel-Strasse 33, 1180 Vienna, Austria; 14 Department of Safety and Quality of Fruit and Vegetables, Max Rubner-Institut, Federal Research Institute of Nutrition and Food, Haid-und-Neu-Strasse 9, 76131 Karlsruhe, Germany; 15 Risk Assessment Strategies, German Federal Institute for Risk Assessment (BfR), Max-Dohrn-Strasse 8-10, 10589 Berlin, Germany; 16 Division for In Vitro Toxicology and Biomedicine, Department of Biology, University of Konstanz, Universitaetsstrasse 10, 78464 Constance, Germany; 17 Department of Toxicology, University of Würzburg, Versbacher Strasse 9, 97078 Würzburg, Germany; 18 Department of Food Chemistry and Toxicology, Faculty of Chemistry, University of Vienna, Währinger Strasse 38-40, 1090 Vienna, Austria; 19 Institute for Nutrition Research and Food Science, Rheinische Friedrich-Wilhelms-University Bonn, Fiedrich-Hirzebruch-Allee 7, 53115 Bonn, Germany; 20 Unit Reproductive and Genetic Toxicology, Federal Institute for Drugs and Medical Devices (BfArM), Kurt-Georg-Kiesinger Allee 3, 53175 Bonn, Germany; 21 Charité–Universitätsmedizin Berlin, Charitéplatz 1, 10117 Berlin, Germany; 22 Max Rubner-Institut, Federal Research Institute of Nutrition and Food, Haid-Und-Neu-Straße 9, 76131 Karlsruhe, Germany; 23 Paul-Ehrlich-Institut, Paul-Ehrlich-Strasse 51-59, 63225 Langen, Germany; 24 Institute of Agricultural and Nutritional Sciences, Martin-Luther-University Halle-Wittenberg, Weinbergweg 22, 06120 Halle (Saale), Germany; 25 Department of Toxicology, Leibniz Research Centre for Working Environment and Human Factors (IfADo), Ardeystr. 67, 44139 Dortmund, Germany

## Abstract

Dietary exposure to N-nitrosamines has recently been assessed by the European Food Safety Authority (EFSA) to result in margins of exposure that are conceived to indicate concern with respect to human health risk. However, evidence from more than half a century of international research shows that N-nitroso compounds (NOC) can also be formed endogenously. In this commentary of the Senate Commission on Food Safety (SKLM) of the German Research Foundation (DFG), the complex metabolic and physiological biokinetics network of nitrate, nitrite and reactive nitrogen species is discussed with emphasis on its influence on endogenous NOC formation. Pioneering approaches to monitor endogenous NOC have been based on steady-state levels of *N*-nitrosodimethylamine (NDMA) in human blood and on DNA adduct levels in blood cells. Further NOC have not been considered yet to a comparable extent, although their generation from endogenous or exogenous precursors is to be expected. The evidence available to date indicates that endogenous NDMA exposure could exceed dietary exposure by about 2–3 orders of magnitude. These findings require consolidation by refined toxicokinetics and DNA adduct monitoring data to achieve a credible and comprehensive human health risk assessment.

## Introduction

The EFSA Panel on Contaminants in the Food Chain (CONTAM Panel) recently published a scientific opinion on the human health risks related to the presence of carcinogenic *N*-nitrosamines (*N*-NAs), a well-known group of *N*-nitroso compounds (NOC), in food (EFSA 2023). The opinion evaluated the toxicity of *N*-NAs to animals and humans, estimated the dietary exposure of the European Union (EU) population to *N*-NAs and assessed the human health risks to the EU population due to the estimated dietary exposure to 10 carcinogenic *N*-NAs occurring in food (TCNA).

The EFSA opinion exclusively focused on the risk assessment of human nutritional exposure to pre-formed *N*-NAs in food. Although it was mentioned that measurable NOC levels of unknown origin have been reported in blood, gastric juice, urine and milk, and that their endogenous formation could not be excluded, the potential consequences for risk assessment due to endogenous NOC formation were not taken into consideration.

The SKLM underlines that a comprehensive risk assessment of human exposure to NOC should not disregard the risk exerted by endogenous exposure to these compounds, which are known to be easily formed in the human body.

## Risk assessment: exogenous exposure

Most genotoxic *N*-NAs undergo CYP-mediated oxidation as key event of bioactivation, leading to the formation of alkyldiazonium ions that alkylate nucleophilic sites of biopolymers. Reaction with DNA results in alkylation of DNA bases and the polydeoxyribonucleotide backbone, yielding DNA base and phosphotriester adducts. DNA base adducts at oxygen sites (e.g., the *O*^6^ position of guanine or the *O*^4^ position of thymine) represent promutagenic lesions that, if unrepaired, cause miscoding and heritable mutations (EFSA 2023). For instance, *O*^6^-alkyl-guanine adducts generate G > A transition mutations and can initiate malignant cell transformation and carcinogenesis. Tumors can be induced by NOC in practically every tissue in a wide spectrum of species up to subhuman primates, with no species having been found to be resistant to NOC carcinogenicity up to now. In rodents, the liver is the main target tissue for the carcinogenic activity of *N*-NAs, followed by the upper gastrointestinal and respiratory tract (EFSA 2023). The key mode of action underlying the carcinogenic activity of *N*-NAs is genotoxicity.

For substances that are both genotoxic and carcinogenic, the EFSA Scientific Committee stated that a margin of exposure (MOE) of 10,000 or higher, if based on the BMDL_10_ from an animal carcinogenicity study, would be of low concern from a public health point of view (EFSA 2005). The CONTAM Panel characterized the risk associated with two nutritional scenarios, which led to dietary exposure estimates at the 95th percentile (P95) of about 0–0.2 µg/kg bw/day across surveys, age groups and scenarios (EFSA 2023). The resulting MOE for the TCNA, based on the BMDL_10_ of *N*-nitrosodiethylamine (NDEA) of 10 μg/kg bw/day for the increased incidence of liver tumors in rodents (benign and malignant tumors combined), was within a range of 3337 to 48 (EFSA 2023). The CONTAM Panel noted that there were significant sources of uncertainty with respect to the P95 exposure assessment (high number of left censored data, lack of data on important food categories) which could make the true value up to a factor of three times lower or a factor of eight times higher. It was concluded that the MOE for TCNA at the P95 exposure level is highly likely (98–100% certain) to be less than 10,000 for all age groups, thus raising a health concern. However, given that these compounds are also formed endogenously, assessment of the human health risk associated with exposure to NOC needs to comprehensively consider exogenous as well as endogenous exposure. For the latter, it is essential to realize that endogenous formation implies not only NOC themselves but also the relevant precursors of *N*-nitrosating agents, including nitrate (NO_3_^−^), nitrite (NO_2_^−^), nitrogen monoxide (NO) and related nitrosating species (Eisenbrand et al. 2022; Habermeyer et al. 2015).

## Exposure to *N*-nitrosating agents: the complex interrelationship between nitrate, nitrite and nitrogen monoxide

### Exogenous nitrate exposure

Exogenous exposure to nitrate has been assessed by EFSA considering different scenarios (EFSA 2008). A mean dietary nitrate uptake for adults of 157 mg/day was estimated, equivalent to 2.6 mg/kg bw/day (based on a body weight of 60 kg). An acceptable daily intake (ADI) value of 222 mg/day (0–3.7 mg/kg bw/day) was set for nitrate as a food additive (FAO/WHO 2003; JECFA 2002). However, individual consumption habits are known to cause a large interindividual variability of exposure that may lead to uptake levels markedly exceeding the ADI.

**Fig. 1:**
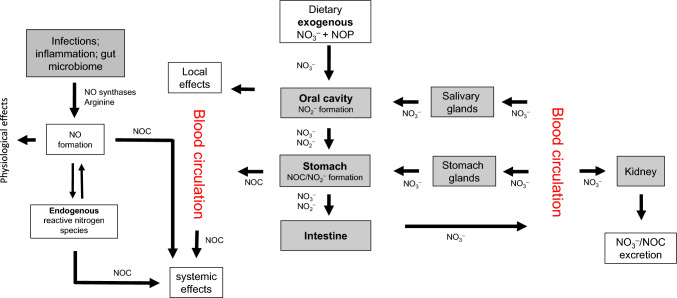
Endogenous exposure to *N*-nitrosating agents: the complex interrelationship between nitrate, nitrite and nitrogen monoxide (adapted from Eisenbrand et al. 2022; Habermeyer et al. 2015). *NO* nitrogen monoxide; *NO*_*2*_^*−*^ nitrite; *NO*_*3*_^*−*^ nitrate; *NOC* N-nitroso compounds; *NOP* nitrosatable precursors

Nitrate ingested with food is rapidly distributed through the blood circulation after absorption from the upper gastrointestinal tract (Fig. 1). When reaching the salivary glands, nitrate is secreted by active transport from blood into saliva, achieving salivary nitrate levels up to 20-times the plasma level. In the oral cavity, salivary nitrate is partially converted into nitrite by oral and commensal microbial reductases (Eisenbrand et al. 2022; Eisenbrand et al. 1980; L’Heureux et al. 2023; Liu et al. 2023). Approximately 25% of the orally ingested nitrate is secreted through the salivary glands and up to 7–8% of the totally ingested nitrate becomes converted to nitrite in the oral cavity during entero-salivary circulation (Spiegelhalder et al. 1976; Tannenbaum et al. 1976; Tricker and Preussmann 1987). It has also been shown that increased salivary nitrite production resulting from nitrate intake enhances oral nitric oxide production in humans (Duncan et al. 1995).

### Exogenous nitrite exposure

Exogenous exposure to nitrite is orders of magnitude lower when compared to nitrate and predominantly due to the presence of residual nitrite in cured meat products, resulting in a mean dietary consumer exposure to nitrites of 5–30 μg/kg bw/ day (adults) and 9–60 μg/kg bw/day (children) in the EU (EFSA 2010). In 2002, JECFA set an ADI of 0–0.07 mg/kg bw for nitrite (JECFA 2002). Nitrite may also be formed from nitrate by chemical and/or microbiological reduction in the environment, during food processing or (inadequate) food storage and, as described below, in the mammalian organism.

### Endogenous nitrate exposure

It is important to note that not only nutritional (exogenous) nitrate exposure but also endogenous exposure is of relevance. In humans, nitrate excreted in urine has been reported to exceed the amount ingested, pointing to an additional exposure by endogenous nitrate biosynthesis at a level of about 10 μmol/kg bw/day, equivalent to about 0.7 mg/kg bw/day or roughly 50 mg/day for a person weighing 70 kg (Green et al. 1981; Tannenbaum et al. 1978). Endogenous nitrate biosynthesis was reported to be markedly increased after endotoxin treatment of experimental animals (Wagner et al. 1983). Activation of mouse macrophages was shown to induce the formation of nitrite and nitrate from their precursor amino acid, L-arginine (Marletta et al. 1988). Furthermore, infections induced by bacteria, parasites or viruses as well as inflammatory diseases, such as gastritis, hepatitis, and colitis have been shown to favor the enhanced biosynthesis of NO (see next chapter), leading to increasing nitrite and nitrate levels (Bartsch et al. 1992; Ohshima et al. 1994; Ohshima and Bartsch 1994; Schaus 1956).

### Endogenous exposure to *N*-nitrosating agents

**Fig. 2:**
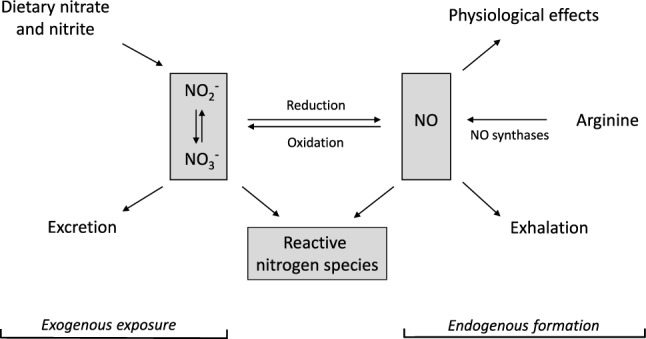
Metabolic interconversion and fate of nitrate (*NO*_3_^–^), nitrite (*NO*_2_^–^) and nitric oxide (*NO*), modified according to (Habermeyer et al. 2015)

The SKLM has extensively reviewed the complex metabolic network between nitrate, nitrite and nitrogen oxides (NO_x_) (SKLM 2014) and has stated that in the mammalian organism, nitrate and nitrite may function as an alternative source for NO, an important and multifaceted physiological signaling molecule, normally generated from arginine by NO synthases (NOS) (Fig. 2). Inflammation is accompanied by an upregulation of inducible nitrogen oxide synthase (i-NOS) in many tissues that can produce NO in excess for a prolonged period of time (Hofseth et al. 2003; Hussain et al. 2008). Formation of nitrite and nitrate was reported to occur through NO generation by NOS. NO in endothelial cells was identified as the endothelium-derived relaxation factor that induces vascular smooth muscle relaxation (Hevel et al. 1991; Palmer et al. 1988). In the mammalian organism, nitrate, nitrite and NO_x_ are metabolically interconvertible.

Of note, although NO itself is not a nitrosating agent and rather short-lived, in the presence of oxygen and/or reactive oxygen species (ROS) it may give rise to N-nitrosating agents, including NO_2_^−^ and NO_x_. Moreover, a variety of biological species such as heme iron proteins and their cognate nitroso complexes may mediate *N*-nitrosation reactions (Jeyakumar et al. 2017; Turesky 2018). In contrast to an acid-catalyzed *N*-nitrosation reaction that primarily occurs in the stomach, such biological *N*-nitrosating species mediate NOC formation at a neutral pH. Likewise, various enteric bacteria have been demonstrated to potently catalyze *N*-nitrosation (Calmels et al. 1985; Kunisaki and Hayashi 1979; Leach et al. 1985; Suzuki and Mitsuoka 1984), and such a catalysis was shown to directly depend on bacterial nitrate reductase(s) (Calmels et al. 1988).

### Endogenous NOC formation

Endogenous NOC formation has primarily been proven to occur in the case of *N*-nitrosatable secondary amino acids such as proline (Knight et al. 1991), hydroxyproline (Ohshima et al. 1982b) and thiazolidine-4-carboxylic acid as well as its congeners (Ohshima et al. 1984). The corresponding NOC are formed from the respective amino acids following nutritional uptake in the upper gastrointestinal tract, especially in the acidic stomach. These NOC are not genotoxic, mutagenic or carcinogenic and are rapidly and almost quantitatively excreted in the urine. These particular NOC have therefore been extensively used to monitor endogenous *N*-nitrosation in the human gastrointestinal tract. In volunteers, ingestion of nitrate has been demonstrated to lead to enhanced urinary excretion of *N*-nitrosated amino acids (Ohshima and Bartsch 1988; Ohshima et al. 1982a; Tricker and Preussmann 1987).

### Formation of NOC in the stomach

Gastric NOC formation from amino compounds primarily occurs in the acidic medium of the stomach and follows well-known *N*-nitrosation kinetics (Fig. 1). Gastric *N*-nitrosation is governed by pH, with optimum rates at about pH 3.4, which corresponds to the pKa value of nitrous acid (HNO_2_). The nitrosation rate slows down at a lower pH because protonation of the *N*-nitrosatable amine is competing with *N*-nitrosation. A similar rate limiting effect occurs at a higher pH, because the concentration of the protonated form of nitrous acid (HNO_2_) from nitrite decreases following the mass action law (1). This entails accordingly reduced availability of the ultimate nitrosating agent, N_2_O_3_ which is formed from two molecules of undissociated HNO_2_ in an acidic medium (2) (Mirvish 1975):

1 $${\text{NO}}_{2}^{ - } + {\text{H}}^{ + } \rightleftharpoons {\text{HNO}}_{2}$$

2 $$2\;{\text{HNO}}_{2} \rightleftharpoons {\text{N}}_{2} {\text{O}}_{3} + {\text{H}}_{2} {\text{O}}$$

### Extragastric NOC formation

In contrast to acid-catalyzed gastric NOC formation, extragastric NOC formation is considered to be independent of an acidic medium, it may occur even more rapidly under neutral or (slightly) basic conditions. Enhanced formation of NOC in the human gastrointestinal tract has been correlated with red meat consumption, suggesting a catalytical role of iron heme complexes as one of several potential causative factors contributing to enhanced colorectal cancer incidence (Bingham et al. 1996). *N*-nitrosation of the amino acid glycine by *N*-nitrosating species has been shown to yield the methylating and carboxymethylating genotoxin diazoacetate. Though rather unstable, diazoacetate has been shown to alkylate DNA, leading to the formation of *O*^6^-methyl- and carboxymethyl-guanine adducts (Shuker and Margison 1997; Shuker 2000).

Depending on the availability of *N*-nitrosatable precursors (NOP), a whole spectrum of putative alkylating genotoxins may arise. Relevant precursors have been shown to comprise not only *N*-nitrosatable food constituents, primarily those bearing primary or secondary amino groups, but many other environmental compounds, foremost certain drug molecules. Many of the resulting NOC have been shown to exert genotoxic, mutagenic, and carcinogenic effects (Eisenbrand 1990).

It thus becomes evident that to comprehensively assess the potential human health risk resulting from the endogenous formation of NOC, dosimetry based alone on *N*-nitrosated amino acids excreted in the urine would be misleading. Moreover, although the monitoring of NOC in appropriate body fluids appears more informative, it may still not cover the full spectrum, especially when highly unstable diazonium intermediates potentially formed from primary amines/amino acids are taken into consideration as well. Thus, to approach a comprehensive risk assessment, complementary methodology needs to be developed. This may be achieved e.g., using appropriate biomarkers that cover the full spectrum of N-nitrosation products potentially formed in vivo, including highly reactive intermediates such as diazoacetate or other products of diazonium ion formation and their reaction products with biopolymers such as DNA bases or other bionucleophiles (Shuker and Margison 1997; Shuker 2000).

### Approaches to monitor endogenous exposure to NOC

The data base on endogenous NOC exposure is clearly not yet sufficient to ensure reliable risk assessment. This may primarily reflect analytical difficulties (besides adequate sensitivity and specificity, the major one being the proven absence of analytical artifacts) as well as the rapid metabolic turnover of most NOC. NDMA, the predominant NOC found in food, has the highest data density concerning animal/human blood levels and, therefore, the available NDMA data have been used to estimate its endogenous formation. Exposure to other NOC is also likely to be primarily endogenous, but the data base is insufficient to make formal estimates (Hrudey et al. 2013).

In a comprehensive analysis of the available evidence, human blood data were considered the least ambiguous estimates of endogenous formation (Hrudey et al. 2013). The authors presented a mean/95th percentile level of endogenous exposure estimates by combining the data from two papers that resulted in a reasonably large number of analyzed individuals (*N* = 58 + 47 = 105) (Dunn et al. 1986; Simenhoff et al. 1982). Further data (Gough et al. 1983) supported the hypothesis that mean values of human NDMA blood levels represented approximate steady-state levels, as they varied little during the day or for periods as long as 3 months (Hrudey et al. 2013). Accordingly, the NDMA level measured in a fasted, unexposed animal was considered to represent the steady-state concentration. Data on the pharmacokinetics of NDMA in monkeys and mice together with literature data for rats, hamsters, rabbits, dogs, and pigs have been used to allometrically deduce a human clearance rate of 3450 mL/min and a distribution volume of 64,800 mL, assuming a body weight of 70 kg (Gombar et al. 1990). Based on these toxicokinetic characteristics, the mean endogenous NDMA formation was estimated to be approximately 900 µg/day (range 100 to about 2500 µg/day; corresponding to 1.4 to 35 μg/kg bw/day assuming a body weight of 71.5 kg for the adult subjects involved) (Hrudey et al. 2013). However, it should be noted that these values only represent the systemic exposure of adults to NDMA and include NDMA formed endogenously and pre-formed NDMA ingested with food or otherwise absorbed, the latter amounts being negligible in comparison. Nevertheless, these data suggest that a large part of human exposure to NDMA (and presumably to NOC in general) may arise from endogenous formation. Moreover, by applying a similar methodology and formulating a simple mathematical model to estimate the order of magnitude of the NDMA flux, based on blood concentrations and assuming a quasi-steady state between formation and metabolism, Tannenbaum estimated that up to 670 µg/day of NDMA can be formed endogenously (Tannenbaum 1980).

Another approach to estimate NDMA exposure has been based on levels of DNA adducts in blood leukocytes. In rats, steady-state levels of *O*^6^-methyl-dG DNA adducts were shown to be linearly dose-related to NDMA exposure (Souliotis et al. 1995). In a Greek population consisting of 36 mothers and their newborns, maternal and cord blood levels of *O*^6^-methyl-dG were determined in leukocytes (Georgiadis et al. 2000). Mean and max *O*^6^-methyl-dG levels were used to identify the corresponding steady-state oral doses in rats required to produce the same adduct levels (Georgiadis et al. 2011, 2000; Souliotis et al. 1995). Mean levels in cord blood were somewhat lower than in maternal blood (45 nmol vs. 56 nmol of *O*^6^-methyl-dG/mol dG, respectively; *p* < 0.05; approx. 0.9 vs. 1.1* O*^6^-methyl-dG/10^8^ nucleotides^1^), but there was a strong correlation between adduct levels in the two compartments (*p* < 0.0001) (Georgiadis et al. 2000). According to the authors, these adduct levels could not be associated with any known source of external nitrosamine exposure. In a second study on 120 maternal and cord blood pairs, *O*^6^-methyl-dG DNA adducts were detected at levels of 0.65 and 0.38 adducts/10^8^ nucleotides (approx. 32.5 and 19 nmol *O*^6^-methyl-dG/mol dG^2^), respectively, in about 70% of the maternal and 50% of the cord blood samples (Georgiadis et al. 2011). The mean values of the second study are slightly lower than those of the first study, which according to the authors could be partly due to the exclusion of smokers from the second study (Hrudey et al. 2013). Assuming similar biokinetics in rats and humans, estimates of endogenous NDMA formation based on *O*^6^-methyl-dG levels in leukocytes resulted in a mean value of 18 µg/kg bw/day (1 360 µg/day total) and a maximum value of 220 µg/kg bw/day (17 000 µg/day total). Thus, despite multiple possible sources of error discussed in detail by Hrudey et al. (2013), the authors concluded that endogenous formation may approach about 1 mg/day and sometimes may be even higher (Hrudey et al. 2013). Of note, pregnant Patas monkeys given orally 100 µg NDMA/kg bw showed adduct levels of 240 nmol *O*^6^-methyl-dG/mol dG (approx. 4.8 *O*^6^-methyl-dG/10^8^ nucleotides^1^) in maternal blood (Chhabra et al. 1995).

These data suggest that the assessment of the health risk of TCNA to humans should be reconsidered. This would require to comprehensively take into account all sources of exposure, foremost the exposure of endogenous origin, which, according to the pioneering estimates presented above, may exceed by far the exposure from dietary intake. Nevertheless, the above-mentioned estimates require confirmation by state-of-the-art analytical methodology and advanced biokinetic modelling, such as recently proposed by (Kang et al. 2024). The overarching aim is to provide a reliable database for a comprehensive risk assessment of human exposure to NOC.

### Research needs

The estimates of endogenous human exposure to methylating NOCs available at present clearly need further corroboration. The remarkably different order of magnitude as compared to the exogenous exposure of 0–0.2 µg/kg bw/day (P95) across surveys and age groups deserves a careful reexamination using well designed state-of-the-art methodology. This should enable a comprehensive risk assessment that certainly needs to take endogenous exposure into closer consideration.

Of note, endogenous exposure relates to a substantial array of further genotoxic agents beyond NOC, as compellingly assessed in a recent comprehensive review (Rietjens et al. 2022). The SKLM concurs with the recommendation expressed by (Rietjens et al. 2022) that regulatory bodies should develop a generally accepted methodology on how to balance risks associated with endogenous exposures against those from exogenous sources.

For NOC, research needs may include:

- Identification of biomarkers and/or methodology for quantification of exposure and discrimination of exogenous versus endogenous sources
- Elucidation of endogenous formation pathways, thereby also considering the human microbiome and its relevance regarding in vivo generation of genotoxic versus nongenotoxic NOC
- Establishment of compound- or group-specific exposure biomarkers
- Building an extended database, based on dependable dosimetry of total exposure, with the aim to achieve probabilistic estimates of total NOC exposure of endogenous versus exogenous origin.
- Determination of the potentially unavoidable endogenous background levels as a reference for the mitigation of exogenous exposure.

Note: The SKLM considers the mitigation measures successfully applied in recent years for potent genotoxic carcinogens to be essential and these should continue to be applied to achieve reduced overall exposure to genotoxic carcinogens.

## Data Availability

All data supporting the findings of this study are available within the paper.

## Footnotes

1 Assuming a content of 20% G in mammalian DNA (Hrudey et al. 2013) and dividing by 5 and then by 10 to convert to a denominator of 10^8^.

2 Assuming a content of 20% G in mammalian DNA (Hrudey et al. 2013) and multiplying by 5 and then by 10 to convert to a denominator of 10^9^.

## References

[CR1] Bartsch H, Ohshima H, Pignatelli B, Calmels S (1992). Endogenously formed N-nitroso compounds and nitrosating agents in human cancer etiology. Pharmacogenetics.

[CR2] Bingham SA, Pignatelli B, Pollock JR (1996). Does increased endogenous formation of N-nitroso compounds in the human colon explain the association between red meat and colon cancer?. Carcinogenesis.

[CR3] Calmels S, Ohshima H, Vincent P, Gounot AM, Bartsch H (1985). Screening of microorganisms for nitrosation catalysis at pH 7 and kinetic studies on nitrosamine formation from secondary amines by *E. coli* strains. Carcinogenesis.

[CR4] Calmels S, Ohshima H, Bartsch H (1988). Nitrosamine formation by denitrifying and non-denitrifying bacteria: implication of nitrite reductase and nitrate reductase in nitrosation catalysis. J Gen Microbiol.

[CR5] Chhabra SK, Souliotis VL, Harbaugh JW (1995). O6-methylguanine DNA adduct formation and modulation by ethanol in placenta and fetal tissues after exposure of pregnant patas monkeys to N-nitrosodimethylamine. Cancer Res.

[CR6] Duncan C, Dougall H, Johnston P (1995). Chemical generation of nitric oxide in the mouth from the enterosalivary circulation of dietary nitrate. Nat Med.

[CR7] Dunn SR, Pensabene JW, Simenhoff ML (1986). Analysis of human blood for volatile N-nitrosamines by gas chromatography-chemiluminescence detection. J Chromatogr.

[CR8] EFSA (2005). Opinion of the Scientific Committee on a request from EFSA related to a harmonised approach for risk assessment of substances which are both genotoxic and carcinogenic. EFSA J.

[CR9] EFSA (2008). Nitrate in vegetables—scientific opinion of the panel on contaminants in the food chain. EFSA J.

[CR10] EFSA (2010). Statement on nitrites in meat products—scientific opinion of the panel on food additives and nutrient sources added to food. EFSA J.

[CR11] Schrenk D, Bignami M, Bodin L, Chipman JK, del Mazo J, Hogstrand C, Hoogenboom L, Leblanc JC, Nebbia CS, EFSA Panel on Contaminants in the Food Chain (EFSA CONTAM Panel) (2023). Risk assessment of N‐nitrosamines in food. EFSA J.

[CR12] Eisenbrand G, Spiegelhalder B, Preussmann R (1980). Nitrate and nitrite in saliva. Oncology.

[CR13] Eisenbrand G, Baum M, Cartus AT (2022). Salivary nitrate/nitrite and acetaldehyde in humans: potential combination effects in the upper gastrointestinal tract and possible consequences for the in vivo formation of N-nitroso compounds-a hypothesis. Arch Toxicol.

[CR14] Eisenbrand G (1990) Endogenous nitrosation–Findings and problems In: Eisenbrand G, Bozler G, von Nicolai H (Eds.), The Significance of N-Nitrosation of Drugs. Gustav Fischer Verlag Stuttgart

[CR15] FAO/WHO (2003) Nitrate (and Potential Endogenous Formation of N-Nitroso Compounds). In: Safety Evaluation of Certain Food Additives and Contaminants, World Health Organization, Joint FAO/WHO Expert committee on Food Additives, Geneva, WHO Food Additives Series No. 50. http://www.inchem.org/documents/jecfa/jecmono/v50je06.htm.

[CR16] Georgiadis P, Samoli E, Kaila S, Katsouyanni K, Kyrtopoulos SA (2000). Ubiquitous presence of O6-methylguanine in human peripheral and cord blood DNA. Cancer Epidemiol Biomarkers Prev.

[CR17] Georgiadis P, Kaila S, Makedonopoulou P (2011). Development and validation of a new, sensitive immunochemical assay for O6-methylguanine in DNA and its application in a population study. Cancer Epidemiol Biomark Prev.

[CR18] Gombar CT, Harrington GW, Pylypiw HM (1990). Interspecies scaling of the pharmacokinetics of N-nitrosodimethylamine. Cancer Res.

[CR19] Gough TA, Webb KS, Swann PF (1983). An examination of human blood for the presence of volatile nitrosamines. Food Chem Toxicol.

[CR20] Green LC, Ruiz de Luzuriaga K, Wagner DA (1981). Nitrate biosynthesis in man. Proc Natl Acad Sci U S A.

[CR21] Habermeyer M, Roth A, Guth S (2015). Nitrate and nitrite in the diet: how to assess their benefit and risk for human health. Mol Nutr Food Res.

[CR22] Hevel JM, White KA, Marletta MA (1991). Purification of the inducible murine macrophage nitric oxide synthase Identification as a flavoprotein. J Biol Chem.

[CR23] Hofseth LJ, Hussain SP, Wogan GN, Harris CC (2003). Nitric oxide in cancer and chemoprevention. Free Radic Biol Med.

[CR24] Hrudey SE, Bull RJ, Cotruvo JA, Paoli G, Wilson M (2013). Drinking water as a proportion of total human exposure to volatile N-nitrosamines. Risk Anal.

[CR25] Hussain SP, He P, Subleski J (2008). Nitric oxide is a key component in inflammation-accelerated tumorigenesis. Cancer Res.

[CR26] JECFA (2002) Evaluation of certain food additives. Fifty‐ninth report of the Joint FAO/WHO Expert Committee on Food Additives. WHO Technical Report Series, no. 913. Geneva, 4–13 June 2002 12677645

[CR27] Jeyakumar A, Dissabandara L, Gopalan V (2017). A critical overview on the biological and molecular features of red and processed meat in colorectal carcinogenesis. J Gastroenterol.

[CR28] Kang DW, Kim JH, Choi GW, Cho SJ, Cho HY (2024). Physiologically-based pharmacokinetic model for evaluating gender-specific exposures of N-nitrosodimethylamine (NDMA). Arch Toxicol.

[CR29] Knight TM, Forman D, Ohshima H, Bartsch H (1991). Endogenous nitrosation of L-proline by dietary-derived nitrate. Nutr Cancer.

[CR30] Kunisaki N, Hayashi M (1979). Formation of N-nitrosamines from seconday animes and nitrite by resting cells of Escherichia coli B. Appl Environ Microbiol.

[CR31] Leach S, Challis B, Cook A, Hill M, Thompson M (1985). Bacterial catalysis of the N-nitrosation of secondary amines. Biochem Soc Trans.

[CR32] L'Heureux JE, van der Giezen M, Winyard PG, Jones AM, Vanhatalo A (2023). Localisation of nitrate-reducing and highly abundant microbial communities in the oral cavity. PLoS ONE.

[CR33] Liu H, Huang Y, Huang M (2023). From nitrate to NO: potential effects of nitrate-reducing bacteria on systemic health and disease. Eur J Med Res.

[CR34] Marletta MA, Yoon PS, Iyengar R, Leaf CD, Wishnok JS (1988). Macrophage oxidation of L-arginine to nitrite and nitrate: nitric oxide is an intermediate. Biochemistry.

[CR35] Mirvish SS (1975). Formation of N-nitroso compounds: chemistry, kinetics, and in vivo occurrence. Toxicol Appl Pharmacol.

[CR36] Ohshima H, Bartsch H (1988). Urinary N-nitrosamino acids as an index of exposure to N-nitroso compounds. IARC Sci Publ.

[CR37] Ohshima H, Bartsch H (1994). Chronic infections and inflammatory processes as cancer risk factors: possible role of nitric oxide in carcinogenesis. Mutat Res.

[CR38] Ohshima H, Bereziat JC, Bartsch H (1982). Measurement of endogenous n-nitrosation in rats and humans by monitoring urinary and faecal excretion of N-nitrosamino acids. IARC Sci Publ.

[CR39] Ohshima H, Bereziat JC, Bartsch H (1982). Monitoring N-nitrosamino acids excreted in the urine and feces of rats as an index for endogenous nitrosation. Carcinogenesis.

[CR40] Ohshima H, O'Neill IK, Friesen M, Bereziat JC, Bartsch H (1984). Occurrence in human urine of new sulphur-containing N-nitrosamino acids N-nitrosothiazolidine 4-carboxylic acid and its 2-methyl derivative, and their formation. J Cancer Res Clin Oncol.

[CR41] Ohshima H, Bandaletova TY, Brouet I (1994). Increased nitrosamine and nitrate biosynthesis mediated by nitric oxide synthase induced in hamsters infected with liver fluke (Opisthorchis viverrini). Carcinogenesis.

[CR42] Palmer RM, Ashton DS, Moncada S (1988). Vascular endothelial cells synthesize nitric oxide from L-arginine. Nature.

[CR43] Rietjens I, Michael A, Bolt HM (2022). The role of endogenous versus exogenous sources in the exposome of putative genotoxins and consequences for risk assessment. Arch Toxicol.

[CR44] Schaus R (1956). Griess' nitrite test in diagnosis of urinary infection. J Am Med Assoc.

[CR45] Shuker DEG, Eisenbrand G (2000). The role of nitrosation: Exogenous vs. endogenous exposure to N-nitroso compounds. Carcinogenic/anticarcinogenic factors in foods: novel concepts.

[CR46] Shuker DE, Margison GP (1997). Nitrosated glycine derivatives as a potential source of O6-methylguanine in DNA. Cancer Res.

[CR47] Simenhoff M, Dunn S, Kirkwood R, Fiddler W, Pensabene J (1982). Presence of nitrosamines in blood of normal and diseased human subjects.

[CR48] SKLM (2014) Opinion on nitrate and nitrite in the diet: an approach to assess benefit and risk for human health. Adopted on April 15th 2014. https://www.dfg.de/resource/blob/171380/e58e7784bfb20c1a8c1f49e08a5c74b4/sklm-opinion-nitrate-nitrite-data.pdf

[CR49] Souliotis VL, Chhabra S, Anderson LM, Kyrtopoulos SA (1995). Dosimetry of O6-methylguanine in rat DNA after low-dose, chronic exposure to N-nitrosodimethylamine (NDMA). Implications for the mechanism of NDMA hepatocarcinogenesis. Carcinogenesis.

[CR50] Spiegelhalder B, Eisenbrand G, Preussmann R (1976). Influence of dietary nitrate on nitrite content of human saliva: possible relevance to in vivo formation of N-nitroso compounds. Food Cosmet Toxicol.

[CR51] Suzuki K, Mitsuoka T (1984). N-nitrosamine formation by intestinal bacteria. IARC Sci Publ.

[CR52] Tannenbaum SR (1980). A model for estimation of human exposure to endogenous N-nitrosodimethylamine. Oncology.

[CR53] Tannenbaum SR, Weisman M, Fett D (1976). The effect of nitrate intake on nitrite formation in human saliva. Food Cosmet Toxicol.

[CR54] Tannenbaum SR, Fett D, Young VR, Land PD, Bruce WR (1978). Nitrite and nitrate are formed by endogenous synthesis in the human intestine. Science.

[CR55] Tricker AR, Preussmann R (1987). Influence of cysteine and nitrate on the endogenous formation of N-nitrosamino acids. Cancer Lett.

[CR56] Turesky RJ (2018). Mechanistic evidence for red meat and processed meat intake and cancer risk: a follow-up on the international agency for research on cancer evaluation of 2015. Chimia (aarau).

[CR57] Wagner DA, Young VR, Tannenbaum SR (1983). Mammalian nitrate biosynthesis: incorporation of 15NH3 into nitrate is enhanced by endotoxin treatment. Proc Natl Acad Sci U S A.

