# Correction: Commentary of the SKLM to the EFSA opinion on risk assessment of N-nitrosamines in food

**DOI:** 10.1007/s00204-024-03776-5

**Published:** 2024-05-23

**Authors:** Gerhard Eisenbrand, Andrea Buettner, Patrick Diel, Bernd Epe, Petra Först, Tilman Grune, Dirk Haller, Volker Heinz, Michael Hellwig, Hans-Ulrich Humpf, Henry Jäger, Sabine Kulling, Alfonso Lampen, Marcel Leist, Angela Mally, Doris Marko, Ute Nöthlings, Elke Röhrdanz, Joachim Spranger, Pablo Steinberg, Stefan Vieths, Wim Wätjen, Jan G. Hengstler

**Affiliations:** 1 Kühler Grund 48/1, 69126 Heidelberg, Germany; 2 Chair of Aroma and Smell Research, Friedrich-Alexander-Universität Erlangen-Nürnberg, Henkestrasse 9, 91054 Erlangen, Germany; 3 Fraunhofer Institute for Process Engineering and Packaging IVV, Giggenhauser Strasse 35, 85354 Freising, Germany; 4 Department of Molecular and Cellular Sports Medicine, Institute of Cardiovascular Research and Sports Medicine, German Sport University Cologne, Am Sportpark Müngersdorf 6, 50933 Cologne, Germany; 5 Institute of Pharmaceutical and Biomedical Sciences, University of Mainz, Staudingerweg, 55128 Mainz, Germany; 6 Food Process Engineering, TUM School of Life Sciences, Technical University of Munich, Weihenstephaner Berg 1, 85354 Freising, Germany; 7 German Institute of Human Nutrition Potsdam-Rehbrücke (DIfE), Arthur-Scheunert-Allee 114-116, 14558 Nuthetal, Germany; 8 Chair of Nutrition and Immunology, Technical University of Munich, Gregor-Mendel-Strasse 2, 85354 Freising, Germany; 9 ZIEL Institute for Food and Health, Technical University of Munich, Weihenstephaner Berg 1, 85354 Freising, Germany; 10 DIL German Institute of Food Technology, Professor-von-Klitzing-Strasse 7, 49610 Quakenbrück, Germany; 11 Chair of Special Food Chemistry, Technical University Dresden, Bergstrasse 66, 01062 Dresden, Germany; 12 Institute of Food Chemistry, University of Münster, Corrensstrasse 45, 48149 Münster, Germany; 13 University of Natural Resources and Life Sciences, Gregor-Mendel-Strasse 33, 1180 Vienna, Austria; 14 Department of Safety and Quality of Fruit and Vegetables, Max Rubner-Institut, Federal Research Institute of Nutrition and Food, Haid-und-Neu-Strasse 9, 76131 Karlsruhe, Germany; 15 Risk Assessment Strategies, German Federal Institute for Risk Assessment (BfR), Max-Dohrn-Strasse 8-10, 10589 Berlin, Germany; 16 Division for In Vitro Toxicology and Biomedicine, Department of Biology, University of Konstanz, Universitätsstrasse 10, 78464 Constance, Germany; 17 Department of Toxicology, University of Würzburg, Versbacher Strasse 9, 97078 Würzburg, Germany; 18 Department of Food Chemistry and Toxicology, Faculty of Chemistry, University of Vienna, Währinger Strasse 38-40, 1090 Vienna, Austria; 19 Institute for Nutrition Research and Food Science, Rheinische Friedrich-Wilhelms-University Bonn, Fiedrich-Hirzebruch-Allee 7, 53115 Bonn, Germany; 20 Unit Reproductive and Genetic Toxicology, Federal Institute for Drugs and Medical Devices (BfArM), Kurt-Georg-Kiesinger Allee 3, 53175 Bonn, Germany; 21 Charité–Universitätsmedizin Berlin, Charitéplatz 1, 10117 Berlin, Germany; 22 Max Rubner-Institut, Federal Research Institute of Nutrition and Food, Haid-Und-Neu-Straße 9, 76131 Karlsruhe, Germany; 23 Paul-Ehrlich-Institut, Paul-Ehrlich-Strasse 51-59, 63225 Langen, Germany; 24 Institute of Agricultural and Nutritional Sciences, Martin-Luther-University Halle-Wittenberg, Weinbergweg 22, 06120 Halle (Saale), Germany; 25 Department of Toxicology, Leibniz Research Centre for Working Environment and Human Factors (IfADo), Ardeystr. 67, 44139 Dortmund, Germany

**Correction: Archives of Toxicology ** 10.1007/s00204-024-03726-1

The article Commentary of the SKLM to the EFSA opinion on risk assessment of N-nitrosamines in food, written by Gerhard Eisenbrand, Andrea Buettner, Patrick Diel, Bernd Epe, Petra Först, Tilman Grune, Dirk Haller, Volker Heinz, Michael Hellwig, Hans-Ulrich Humpf, Henry Jäger, Sabine Kulling, Alfonso Lampen, Marcel Leist, Angela Mally, Doris Marko, Ute Nöthlings, Elke Röhrdanz, Joachim Spranger, Pablo Steinberg, Stefan Vieths, Wim Wätjen, Jan G. Hengstler was originally published electronically on the publisher’s internet portal on 6th March, 2024 without open access. With the author(s)’ decision to opt for Open Choice the copyright of the article changed on 17th April, 2024 to © The Author(s) name in OA Team’s request] 2024 and the article is forthwith distributed under a Creative Commons Attribution 4.0 International License, which permits use, sharing, adaptation, distribution and reproduction in any medium or format, as long as you give appropriate credit to the original author(s) and the source, provide a link to the Creative Commons licence, and indicate if changes were made. The images or other third party material in this article are included in the article’s Creative Commons licence, unless indicated otherwise in a credit line to the material. If material is not included in the article’s Creative Commons licence and your intended use is not permitted by statutory regulation or exceeds the permitted use, you will need to obtain permission directly from the copyright holder. To view a copy of this licence, visit http://creativecommons.org/licenses/by/4.0. Open access funding enabled and organized by Projekt DEAL.

